# Delayed Remote Ischemic Postconditioning Improves Long Term Sensory Motor Deficits in a Neonatal Hypoxic Ischemic Rat Model

**DOI:** 10.1371/journal.pone.0090258

**Published:** 2014-02-28

**Authors:** Pradilka N. Drunalini Perera, Qin Hu, Junjia Tang, Li Li, Margaret Barnhart, Desislava M. Doycheva, John H. Zhang, Jiping Tang

**Affiliations:** 1 Department of Physiology & Pharmacology, Loma Linda University School of Medicine, Loma Linda, California, United States of America; 2 Department of Neurosurgery Loma Linda University School of Medicine, Loma Linda, California, United States of America; 3 Department of Anesthesiology, Loma Linda University School of Medicine, Loma Linda, California, United States of America; Temple University School of Medicine, United States of America

## Abstract

**Objective:**

Remote Ischemic Postconditioning (RIPC) is a promising therapeutic intervention wherein a sub-lethal ischemic insult induced in one organ (limb) improves ischemia in an organ distant to it (brain). The main objective of this study was to investigate the long-term functional effects of delayed RIPC in a neonatal hypoxia-ischemia (HI) rat model.

**Method:**

10 day old rat pups were subjected to delayed RIPC treatment and randomized into four groups: 1) Sham, 2) HI induced, 3) HI +24 hr delayed RIPC, and 4) HI +24 hr delayed RIPC with three consecutive daily treatments. Neurobehavioral tests, brain weights, gross and microscopic brain tissue morphologies, and systemic organ weights were evaluated at five weeks post surgery.

**Results:**

HI induced rats performed significantly worse than sham but both groups of delayed RIPC treatment showed improvement of sensory motor functions. Furthermore, compared to the HI induced group, the delayed RIPC treatment groups showed no further detrimental changes on brain tissue, both grossly and morphologically, and no changes on the systemic organ weights.

**Conclusion:**

Delayed RIPC significantly improves long term sensory motor deficits in a neonatal HI rat model. A 24 hr delayed treatment does not significantly attenuate morphological brain injury but does attenuate sensory motor deficits. Sensory motor deficits improve with both a single treatment and with three consecutive daily treatments, and the consecutive treatments are possibly being more beneficial.

## Introduction

Hypoxic Ischemic (HI) brain injury begins with an initial insult and may continue into the recovery period after reperfusion [1], [2]. Reperfusion injury causes damage that with current medical treatment is largely irreversible. It may occur in the prenatal, perinatal or postnatal periods. Interestingly, only the most severe injuries, accounting for only 25% of cases, are diagnosed at birth [2], [3]. Clinically known as Hypoxic Ischemic Encephalopathy (HIE), it is more likely to occur in an immature brain rather than in the brain of a full-term neonate [4]. Damage resulting from hypoxic ischemia may lead to chronic conditions such as cerebral palsy, mental retardation, learning disabilities, and epilepsy [2], [5]. These conditions confer significant challenges to the affected individual, their family, and society. The remaining 75% of cases that consist of mild and moderate HIE may be diagnosed weeks or even months after the initial insult.

Despite extensive research, no treatment or intervention has been found to significantly improve or reverse these injuries, and the medical scientific community continues to search for therapeutic strategies which might at least minimize injury and improve behavioral function. Treatment using remote ischemic postconditioning (RIPC) can be either rapid or delayed; the former is applied immediately or within a few minutes after reperfusion [6] while delayed treatment is applied hours to weeks after reperfusion. Since rapid RIPC is only applicable for patients with HIE diagnosed immediately after birth, there exists an extremely short therapeutic time window which may make clinical application challenging [6]. In contrast, delayed RIPC (dRIPC) can be initiated on patients presenting anytime beyond few hours and may represent a practical new strategic intervention for HIE with delayed presentations.

The aim of this study is to determine if dRIPC is effective when applied to a neonatal HI rat model, through observation of 1) long term neuro-functional effects, 2) effect on brain volume and weight and 3) effect on systemic organ weights. Our interventions included a single treatment 24 hrs following the injury and three consecutive daily treatments following a 24 hr delay following the injury. Both groups were evaluated after five weeks to assess long term effects. We hypothesize that dRIPC applied 24 hrs after HI will improve long term sensorimotor deficits.

## Materials and Methods

### Ethics Statement

The Institutional Animal Care and Use Committee of Loma Linda University approved all the procedures and protocols in this neonatal HI rat model. Handing of animals was carried out in accordance with National Institute of Health Guide for the Care and Use of Laboratory Animals.

### Animal model/Surgical procedure

Pregnant Sprague-Dawley rat litters with 12–13 pups per litter were obtained from Harlan Laboratories in Livermore, CA. A total of 37 pups were housed along with their dams and provided with food and water *ad libitum*. Due to the inability to determine gender in neonatal rats, unsexed pups were randomized into the following groups: Sham (n = 9), HI induced (n = 9), HI +24 hr dRIPC treatment (n = 9) and HI +24 hr delayed RIPC with three consecutive daily treatments (n = 7). Three pups died during surgery or in the hypoxic chamber, resulting in a mortality rate of 8.1%.

The Rice–Vannucci neonatal HI model was used in this study [7]. Surgery was performed under inhaled isoflurane anesthesia and all efforts were made to minimize suffering. 10 day old rat pups were placed on a 37°C temperature-regulated surgical table, induced with 3% isoflurane (mixed with air and oxygen) and maintained during surgery with 1.5% isoflurane. Using sterile technique, a small midline incision was made on the anterior neck followed by a gentle blunt dissection to identify the right common carotid artery. Once isolated and separated from its surrounding structures the artery was ligated and transected. The sham group of rat pups underwent the same blunt dissection and right carotid artery isolation but no ligation or transection was done. Hemostasis was maintained throughout the procedure, and following the procedure the neck incision was sutured. The total surgical time including induction of anesthesia did not exceed 9 minutes. Rat pups were given a temperature regulated recovery period of 1 hr, then placed in a low-oxygen environment created using an air-tight glass chamber which was submerged in a 37°C water bath in which an 8% Oxygen and 92% nitrogen ambient environment was maintained. The pups were kept in this hypoxic chamber for 2.5 hrs and then returned to their dams. The dams and pups were then held in a 37°C incubator until all treatment procedures were complete.

### Remote Ischemic Post-Conditioning (RIPC) treatment

RIPC consisted of four 10 minute cycles of hind limb ischemia and reperfusion in non-anesthetized unrestrained rat pups as previously described [8]. To produce limb ischemia, two ends of a cut rubber band were passed through a small rubber tube to form a reversible snare. Ischemia was induced by encircling this snare around each hind limb of the rat pups, and clamped using hemostatic forceps for 10 minutes. The clamp and rubber band were then released to allow reperfusion for 10 minutes. This was repeated four times. Limb ischemia was confirmed with observation of swelling and cyanosis of the limbs, and reperfusion was confirmed by return of the limbs to their original pink color.

RIPC was initiated 24 hrs after HI surgery for two treatment groups. The first group received a single treatment at 24 hrs, and the second group received three consecutive daily treatments: at 24 hrs, 48 hrs and 72 hrs. Following RIPC, animals were closely observed and all pups resumed their normal activities including walking, feeding and sleeping.

### Neurobehavioral Tests

The following neurobehavioral tests were done in a blinded manner at five weeks after the HI.

#### Foot Fault

Tests motor coordination [9]. Rats were placed on a horizontal grid floor elevated above the surface and allowed to walk for 2 minutes. A foot fault was noted when the rat's foot miss–stepped on the grid and the foot fell downwards through the opening between the grids. All four limbs were observed for misses. The total number of left and right foot faults was recorded.

#### Modified Grip Traction (Wire Hang)

A test to evaluate muscle strength [10]. Rats were allowed to hang for a maximum of 60 seconds on a horizontal rope by their forepaws and the time taken to fall was recorded.

#### T maze

A test to ascertain short-term or working memory, as well as complex cortical function [11]. Rats were placed at the base of a T shaped platform in a dark environment and were allowed to explore it until they chose to turn into one of the arms of the maze. Each animal repeated this ten times and the sequence of right and left arm choices were expressed as a percentage of spontaneous alternation.

#### Water Maze

A test of spatial learning and memory [12], [13]. In this test rats attempted to find a hidden (submerged) platform in a pool of water using visual cues. Three trials were conducted on each of four consecutive days. All trials lasted a maximum of 60 seconds. The “cued” trials provide a visible platform above the water level where the rats were allowed to sit for 10 seconds after finding it or being guided to it. For the “spatial” trials, the platform was submerged under water and rats were released and allowed to swim in search of the platform. In this trial the time taken to find the platform over consecutive days represents spatial learning. For the “probe” trial, the platform was removed completely and rats were allowed to swim in search of the platform.

#### Beam Balance

A test of sensorimotor function. Rats were placed with all four limbs onto a stationary horizontal balance beam (50 cm long, 5 cm diameter) for a maximum of 60 seconds and the length walked without falling was recorded (with a 20 cm cut-off in either direction). Scores were assigned as follows: 4 =  walked beyond 20 cm, 3 =  walked<20 cm, 2 =  walked some length but fell, 1 =  could not walk, but remained on balance beam, and 0 =  could not walk, could not remain on beam.

### Evaluation of brain damage and systemic organ weight

The exclusive ipsilateral brain damage that occurs in HI model [14], is best evaluated for long term studies by hemispheric weight loss and been used as the primary variable to estimate brain damage in neonatal HI rats [8], [15], [16].

At five weeks after HI, the rats were euthanized and organs including brain, lung, liver, spleen and heart were harvested. Rats weren't perfused as it would interfere with accuracy in gross organ weights. The brain samples were assessed for gross tissue loss, after which the cerebellum and brainstem were removed. The brains were then divided into left and right hemispheres and weighed on a high-precision balance (sensitivity - 0.001 g). The results were expressed as the mass ratio of the ipsilateral (right) hemisphere compared with the contralateral (left) hemisphere. Gross weights of lung, liver, and spleen were also measured. Weights were expressed as the ratio of organ weight to body weight.

### Evaluation of Brain Morphology

Brain tissue damage (microscopic tissue loss) was assessed and hippocampal (CA1) region was evaluated for neuronal density. The separated brain hemispheres were immersed in phosphate-buffered formalin (PBF) filled tubes and were stored for one week to allow for adequate tissue saturation. The brains were then removed from PBS, immersed in a 30% sucrose solution and stored for three days until they settled at the bottom of the containers, indicating adequate dehydration. Immediately following removal from the sucrose solution, the brain tissue was cryoprotected and rapidly frozen in liquid nitrogen.

Coronal brain sections with 10 µm thickness at the level of the hippocampus were prepared using a cryostat. Nissl staining was performed as previously described [17]. The sections were observed under a light microscope (up to 20× magnification). Brain tissue loss was measured and the morphology of hippocampal CA1 cells was observed by staining. Sections of each experimental group corresponded to Plate 28 of the rat brain atlas [18].

For Nissl quantification of brain atrophy, the residual volume was measured from three Nissl-stained coronal brain sections (front, middle and back) per animal, photographed under light microscopy and analyzed using Image J software (Version 1.46, National Institutes of Health,Bethesda, MD) as previously described [19], [20]. Infarct volume was expressed as a volume percentage by the following formula: (contralateral volume-ipsilateral volume/contralateral volume) ×100%.

### Immunofluorescence staining

Immunofluorescent staining for brain tissue was performed on fixed frozen ultrathin sections as previously described [21], [35]. Slices were incubated with primary mouse anti-NeuN antibodies (diluted 1∶200, CHEMICON International, CA). Hippocampus in the ipsilateral hemisphere of the brain coronal section was chosen and imaged.

### Statistical Analysis

All data were expressed as mean ± SEM. Statistical differences between groups were analyzed with one-way ANOVA followed by Tukey multiple-comparison post-hoc analysis or Student–Newman–Keuls test on ranks and Kruskal-Wallis one way analysis of variance by ranks (a non-parametric analysis) followed by post hoc analysis. A *p* value of <0.05 was considered statistically significant.

## Results

### Delayed RIPC Confers Long Term Protection against HI Induced Neurobehavioral Deficits

All neurobehavioral tests showed that HI induced rats performed significantly worse than the sham group (*p*<0.05) except in the balance beam test which showed a *p* = 0.056.

**Figure 1:**
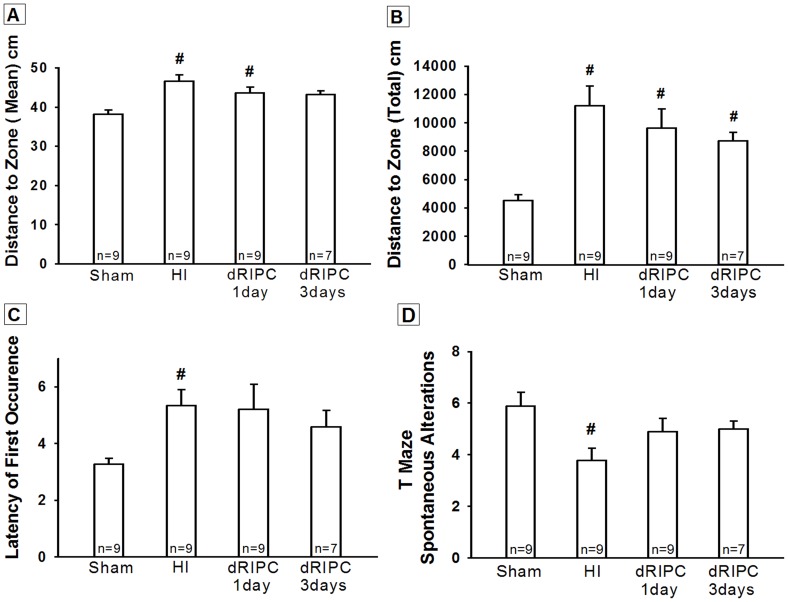
Neurobehavioral tests for cognitive function at five weeks after hypoxia ischemia. All animals in hypoxia ischemia (HI) group exhibited significant cognitive impairment. However, following delayed remote ischemic postconditioning (dRIPC) treatment, a statistical significance was not reached. Nevertheless treatment groups showed improvement, indicated by a consistent trend to reverse the neurobehavioral deficits caused by HI. Morris Water Maze test shows, three day treatment group had improved performance **(A, C)** and a trend of improvement **(B)**, and also one day treatment group shows an improvement in performance **(C)**. The T Maze test **(D)** demonstrates improved functional outcome following dRIPC in both treatment groups. Data expressed as mean ± SEM. #*p*<0.05 compared with sham. *p* = 0.13 vs. HI and 3days and *p* = 0.3 vs. HI and 1day.

The Morris Water Maze test results are shown in Fig 1A, 1B and 1C, and significant differences were shown in all HI groups (*p*<0.05 vs. Sham). Although Fig 1B shows a tendency, no significant difference was noted among treated dRIPC groups (*p* = 0.13 vs. HI and dRIPC - 3days). Fig 1A and 1C show that the damage caused by HI was significantly improved by dRIPC (*p* = 0.07 vs. Sham in Fig 1A (dRIPC - 3days) and 1C).

The T-maze test also demonstrated impairment in the HI group (*p*<0.05 vs. Sham). Both the RIPC treatment groups showed improvement of this neurobehavioral deficit and an improvement in exploratory behavior and short-term memory (*p* = 0.087 vs. Sham, Fig 1D).

**Figure 2:**
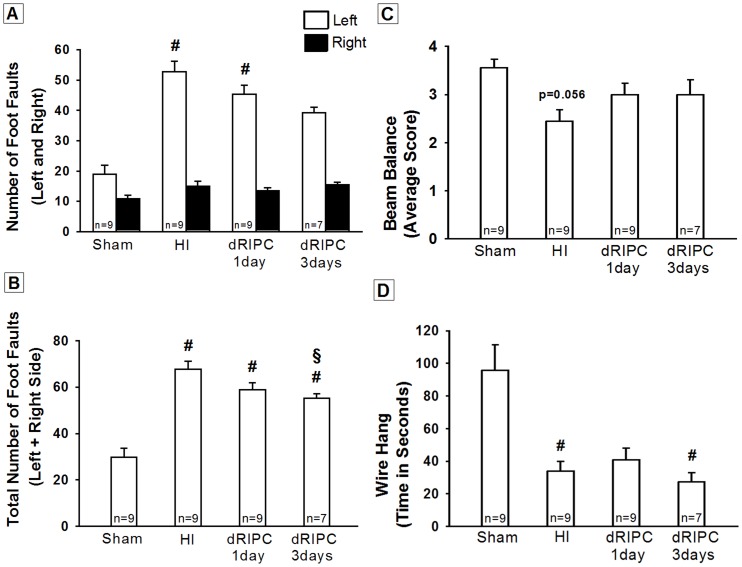
Neurobehavioral tests for sensory motor function at five weeks after hypoxia ischemia. All animals in hypoxia ischemia (HI) group exhibited significant sensory motor impairment. The three day delayed remote ischemic postconditioning treatment group showed a trend to decrease the number of left foot faults after HI **(A)**, and the number of total foot faults was significantly less in the three day treatment group compared to the non-treated HI group **(B)**. The Beam Balance test did not show significant difference between the groups **(C)**. The Wire Hang test did not show significant difference between the treated and non-treated groups **(D)**. Data expressed as mean ± SEM. #*p*<0.05 compared with sham, §*p*<0.05 compared with non-treated HI group.

In the Foot Fault test, the HI group showed significant reduction in muscle strength and motor coordination in their left-side fore and hind limbs (*p*<0.05 vs. Sham, Fig 2A and 2B). The three day treatment group showed a significant improvement of this motor deficit (*p* = 0.085 vs. Sham, Fig 2A and *p*<0.05 vs. Sham but also *p*< 0.05 vs. HI group, Fig 2B).

The beam balance test failed to show a statistical difference between the HI and sham groups, but a strong tendency was observed (*p* = 0.056, Fig 2C). No differences in the balance beam test were found among the treated dRIPC groups.

The Wire Hang test showed a significant difference between the Sham and HI groups (*p*<0.05, Fig 2D) but no therapeutic effect of RIPC was observed.

### Delayed RIPC Shows No Long Term Improvement in Brain and Systemic Organ Weights

**Figure 3:**
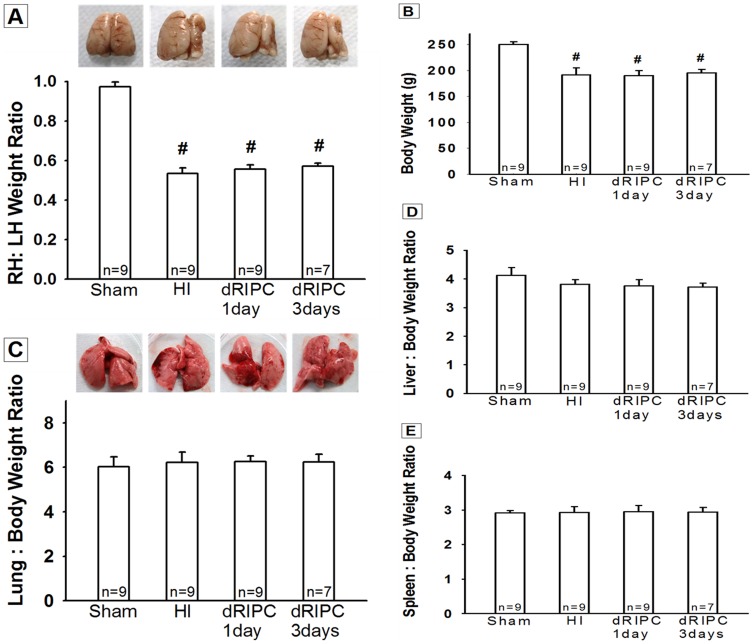
Brain Weight (A), Body Weight (B), and Organ Weights (C), (D), and (E) at five weeks after hypoxia ischemia. **(A)** A significant loss of right-to-left hemispheric (RH: LH) weight ratio was evident after hypoxia ischemia. Although a slight preservation of the injured hemisphere was observed grossly in the delayed remote ischemic postconditioning (dRIPC) treatment groups, there was no significant improvement or worsening of brain weights in both one day and three day treatment groups. Representative pictures of brain samples shown. **(B)** All treated and non-treated groups had a significant loss in body weight compared to sham. dRIPC did not aggravate weight loss. **(C)** Lung, **(D)** Liver, and **(E)** Spleen, the organ: body weight ratios, showed no significant difference between the groups. dRIPC did not adversely affect organ: body weight ratios. The treatment groups showed a slight decrease in pulmonary haemorrhage **(C)**. Representative pictures of lung samples shown. Data expressed as mean ± SEM. #*p*<0.05 compared with sham. *p* = 0.98 vs. RIPC groups and HI in **(B)**.

At 5 weeks after HI, a significant atrophy of ipsilateral brain tissue was observed (*p*<0.05 vs. Sham) and no improvement (grossly) was noted in either post-treatment groups (Fig 3A). Fig 3B shows the long term correlation of physical development and body weights at five weeks. dRIPC failed to improve physical development when compared to the HI group (*p* = 0.98). There was no significant weight gain following dRIPC.

The effects of post-hypoxic injury treatment were explored across multiple organ systems (Fig 3C, 3D and 3E) but treatment groups showed no significant organ weight gain or loss thereby providing adequate information that dRIPC has no detrimental effects to body and is a safe treatment option.

### Delayed RIPC Shows No Changes in Brain Morphology

**Figure 4:**
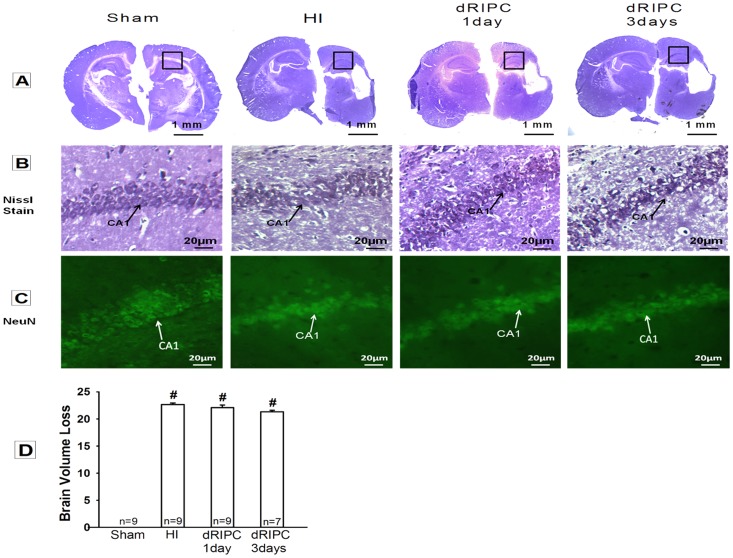
Nissl and immunostaining of NeuN for brain morphology and hippocampal CA1 region at five weeks after hypoxia ischemia. **(A)** All groups except sham showed brain atrophy with loss of brain tissue in the hypoxia ischemia (HI) injured hemisphere. Delayed remote ischemic Postconditioning (dRIPC) did not attenuate or worsen the atrophy. **(B) Nissl and (C) NeuN** Higher magnification of the hippocampus CA1 region (depicted within boxes in **(A)**) showed a decrease of the CA1 cells after HI compared to sham group. dRIPC treatment did not show improvement microscopically. **(D)** Nissl quantification of brain atrophy shows significant brain volume loss in HI. dRIPC groups did not show significant improvement but a trend is seen. Data expressed as mean ± SEM. #*p*<0.05 compared with sham.

Nissl histology of the brain at five weeks post-injury showed brain atrophy in all of the groups except sham (Fig 4A), as demonstrated by vacuolization, neuronal loss and tissue breakdown. Morphology studies showed that dRIPC did not attenuate the brain damage associated with HI. Nissl quantification of brain volume loss confirms the statistical significance between sham and HI group (*p*<0.05, Fig 4D). Treatment groups did not show a significant difference as compared with HI (*p* = 1.18 vs. dRIPC - 3days) but a trend following dRIPC. An enlarged Nissl image shows the comparison of the CA1 cell morphology between all of the groups (Fig 4B) with significant distortion of the CA1 cells, in all HI injured groups in comparison with sham. The NeuN staining, a specific neuron marker also shows the significant distortion CA1 cells in the hippocampus in all HI injured groups as compared with Sham with no morphologic improvement following dRIPC (Fig 4C).

## Discussion

RIPC is an experimental intervention which has the potential to contribute to neural protection following ischemic insult. However, RIPC and its fundamental biology are not well understood, and studies to date are largely observational [22], [23], [24], [34]. Loukogeorgakis et al demonstrated for the first time in humans that RIPC can be induced by transient limb ischemia and provides protection from the effects of acute ischemic brain injury [25]. Multiple subsequent studies also have shown beneficial neuroprotective effects of RIPC in adult cerebral ischemic models [26], [27], [28]. Zhou et al first applied RIPC in a rat neonatal HI model and showed that rapid RIPC immediately following HI significantly reduced the infarct volume and improved neurologic effects [8]. Wei et al showed that repeated limb RIPC provides dose-dependent protection against adverse left ventricular remodeling and improves survival in a rat model of myocardial infarction [29]. Additionally, Ren et al have shown that immediate RIPC and dRIPC provide protection against focal cerebral ischemia in adult rats and improves neurologic function continuing two months after an ischemic event, suggesting long-term neuroprotection [30].

In this study we investigate the potential for long-term neuroprotection using dRIPC in a well-studied neonatal rat model of unilateral hypoxic-ischemic brain injury. We confirmed the following in HI induced rat pups: significant gross brain injury (Fig 3A), a significant decrease in cognitive, sensory and motor functions (Fig 1 and 2), and marked destruction to hippocampal CA1 cells (Fig 4B, 4C), all consistent with other rat neonatal studies that used the Rice-Vannucci neonatal HI model [6], [8], [9], [16], [17], [31], [32]. Our results show that 24 hr dRIPC improves long-term neurobehavioral deficits (Fig 1 and 2) and does not affect systemic organ weights (Fig 3B–3E). dRIPC does not attenuate brain atrophy, shown both grossly and microscopically with no further disruption of hippocampal CA1 cells (Fig 3A, 4A–4D). Hippocampus CA1 cells are more involved in cognitive as compared to motor function [33]. Interestingly, our study shows an improved cognitive deficit caused by HI behaviorally (Fig 1A, 1C) but no morphologic differences seen following dRIPC (Fig 4B, 4C). We were able to show long-term and dose-dependent improvement of sensory, motor, and cognitive impairments with treatment up to five weeks following HI. Single treatments as well as three consecutive daily treatments were shown to be beneficial with consecutive treatment possibly being of greater benefit (Fig 2B).

RIPC is a new experimented non-pharmacologic intervention and is an important therapeutic strategy that can be quickly and easily administered to protect the brain from an ischemic brain insult. The technique of RIPC is simple and can be performed by placing a tourniquet or an inflating a blood pressure cuff for brief periods of time. One of the advantages of this study is that we used hind limbs as the remote organ to induce brief episodes of ischemia, which is a more practical model, as upper limbs are more commonly used for intravenous administrating of fluids and medications. To our knowledge, our data are the first to demonstrate the therapeutic effects of dRIPC on an HI-induced neonatal rat model. Another advantage of this study is that a 24 hr delayed treatment is protective. Our study indicates a neurobehavioral improvement as well as a non-worsening brain injury both grossly (Fig 3A) and microscopically with no further disruption of hippocampal CA1 cells (Fig 4B, 4C). This observation suggests that RIPC is a safe and easy to use therapeutic intervention that can be used as late as 24 hrs after the initial brain injury. We do not have a solid explanation that 24 hr dRIPC improved long term neurobehavioral deficits but did not significantly reduce brain tissue loss (Fig 4D), but to acknowledge that infarction size alone may not reflect the protective effect of a neuroprotectant [30]. The third advantage is that dRIPC has long term benefit effects. We observed in this study that a delayed treatment of dRIPC improved neurological function at five weeks after the initial injury. We noticed that dRIPC has not been studied previously in a neonatal HI model. Future studies are needed to address potential molecular signaling pathways and the potential cause that dRIPC did not reduce brain atrophy but still improved neurological functions at five weeks after HI.

Even though three consecutive treatments may be more effective, more consecutive treatment plans such as five or even ten treatments were not used because experimentally RIPC is distressful for the neonatal limb to handle the ischemic cycles without anesthesia. This may be a potential disadvantage in patient care since distress will affect new born children and it may be difficult to obtain cooperation even though RIPC is a convenient, non invasive and all most pain free intervention.

In conclusion, a 24 hr delayed RIPC significantly improved long term sensory motor deficits in a neonatal rat model even five weeks after HI and three consecutive daily treatments seem being more beneficial. The results highlight dRIPC to be beneficial in neonates who have been diagnosed as late as 24 hrs following an ischemic brain injury by promoting a long term well being and alleviating its severe neurobehavioral deficits. Hence this study mandates further replication, mechanistic and clinical studies to be performed in order to promote this intervention as a bedside clinical practice.
